# Role of HVR1 sequence similarity in the cross-genotypic neutralization of HCV

**DOI:** 10.1186/s12985-020-01408-9

**Published:** 2020-09-18

**Authors:** Alexander I. Mosa, Mounir G. AbouHaidar, Richard A. Urbanowicz, John E. Tavis, Jonathan K. Ball, Jordan J. Feld

**Affiliations:** 1 Department of Cell and Systems Biology, University of Toronto, Toronto, Canada; 2 Wolfson Centre for Global Virus Infections, University of Nottingham, Nottingham, UK; 3 School of Life Sciences, University of Nottingham, Nottingham, UK; 4 Department of Molecular Microbiology and Immunology, Saint Louis University School of Medicine, St. Louis, USA; 5 Toronto Centre for Liver Disease, Toronto General Hospital, Sandra Rotman Centre for Global Health, University of Toronto, Toronto, Canada

**Keywords:** Hypervariable epitope, Cross-reactivity, HCV, Antigenic convergence

## Abstract

Despite available treatments, a prophylactic HCV vaccine is needed to achieve elimination targets. HCV vaccine development has faltered largely because the extreme diversity of the virus limits the protective breadth of vaccine elicited antibodies. It is believed that the principle neutralizing epitope in natural infection, HVR1, which is the most variable epitope in HCV, mediates humoral immune escape. So far, efforts to circumvent HVR1 interference in the induction and function of conserved targeting Ab have failed. Efforts to understand factors contributing to cross-neutralization of HVR1 variants have also been limited. Here, following mouse immunizations with two patient-derived HVR1 peptides, we observe cross-genotype neutralization of variants differing at 15/21 positions. Surprisingly, sequence similarity was not associated with cross-neutralization. It appeared neutralization sensitivity was an intrinsic feature of each variant, rather than emergent from the immunogen specific Ab response. These findings provide novel insight into HVR1-mediated immune evasion, with important implications for HCV vaccine design.

## Introduction

Chronic infection with hepatitis C virus (HCV) is a leading cause of liver disease, cirrhosis, and hepatocellular carcinoma, resulting in 475,000 deaths annually [1]. Estimates of prevalence based on seropositivity range between 1.3 and 2.1%, or between 92 and 149 million individuals globally [2]. Though direct-acting antiviral (DAA) therapy is largely curative, only a minority of chronic infections (~ 20%) have been diagnosed, with even fewer treated (~ 3%) [1]. Persistent challenges in screening, diagnosis, access to affordable DAA, and the risk for re-infection in vulnerable populations aggravates elimination efforts [2]. A prophylactic vaccine for HCV is therefore still urgently needed.

The extreme diversity of the virus, with billions of related but distinct variants circulating in each infected person, has been a major barrier to vaccine development [3]. Early candidate vaccines using mammalian expressed E2 glycoprotein (E2), a meditator of viral entry expressed on the surface of mature virions, were successful in inducing protective immunity in chimpanzee against homologous challenge [4]. However, progression to chronic infection was observed following heterologous challenge, with escape mutations subsequently mapped to the highly variable N-terminus of E2, termed HVR1 [4, 5]. Since then, cohort and in vitro analysis has consistently identified HVR1 as the principle target of neutralizing antibodies (nAb) in natural infection [6].

Recently, the immunodominant HVR1 has been hypothesized to divert the humoral response from conserved neutralizing epitopes [7, 8]. Some groups have therefore sought to elicit cross-neutralizing Ab targeting conserved epitopes by amputating HVR1 from E2 immunogens [9]. It was shown that HVR1-deleted E2 was an inferior immunogen, failing to elicit even homologous nAb following immunization [9]. Variations in HVR1 have since been implicated in resistance to *extra HVR1 targeting neutralizing antibodies,* underscoring the crucial role of the anti-HVR1 response in any potential HCV vaccine [10]. To understand how HCV variation mediates immune escape, global networks of HVR1 cross-reactivity have been elaborated [11]. Consistent with cohort analysis, chimpanzee vaccination, and in vitro neutralization assays, the sequence similarity between two HVR1 peptides was predictive of cross-reactivity [5, 6, 11]. However, cross-reactive pairs with low sequence similarity were also observed, indicating a more complex relationship between HVR1 variability and immune evasion [11]. Moreover, these studies have not established if the observed association between cross-reactivity and sequence similarity applies to cross-neutralization. Given the critical implications of these questions for HCV vaccine design, we sought to clarify these dynamics by synthesizing high-Hamming distance HVR1 peptides, immunizing mice, and evaluating how sequence similarity associated with cross-neutralization. We hypothesized that intrinsic physicochemical features of HVR1 sequences contributing to secondary structure might influence resistance to neutralization.

## Materials and methods

HVR1 shannon variability was mapped by inputting a reference alignment of AA 390–410, obtained from The Los Alamos Hepatitis C Sequence Database, into the Protein Variability server [12, 13]. The patient-derived amplicons used to develop the clonal library from which immunogens I.1 and I.2 were selected has been previously described [14]. HVR1 sequences were synthesized into peptides using Fmoc chemistry, conjugated to keyhole limpet hemocyanin via maleimide linkage, and mixed at 1:1 ratio with Freunds complete or incomplete adjuvant (primary/booster). Mice were subcutaneously injected (35 μg peptide + 35 μL adjuvant) at days 0, 28, and 38, with terminal bleed via cardiac puncture at day 48 [4 female, 4–6 weeks old Balb/c per group—protocol approved by University Health Network (UHN) Animal Care Committee (ACC)]. Mock immunization used adjuvant with sterile PBS. ELISA and neutralization assays were performed as previously described, using heat-inactivated, group pooled sera at the indicated dilution [9]. For physicochemical analysis, the program CRASP was used to transform HVR1 sequences into values representing secondary structure (HELIXF2), based on factor analysis [15]. For Hamming Distance and HELIXF2, statistical analysis was done using linear regression (*P < 0.001). Statistical analysis of neutralization assays and ELISA was done by unpaired *t *test followed by a Benjamini–Hochberg false discovery rate (FDR) adjustment for multiple comparisons (Q = 0.05) using Prism8 [16].

## Findings

**Fig. 1:**
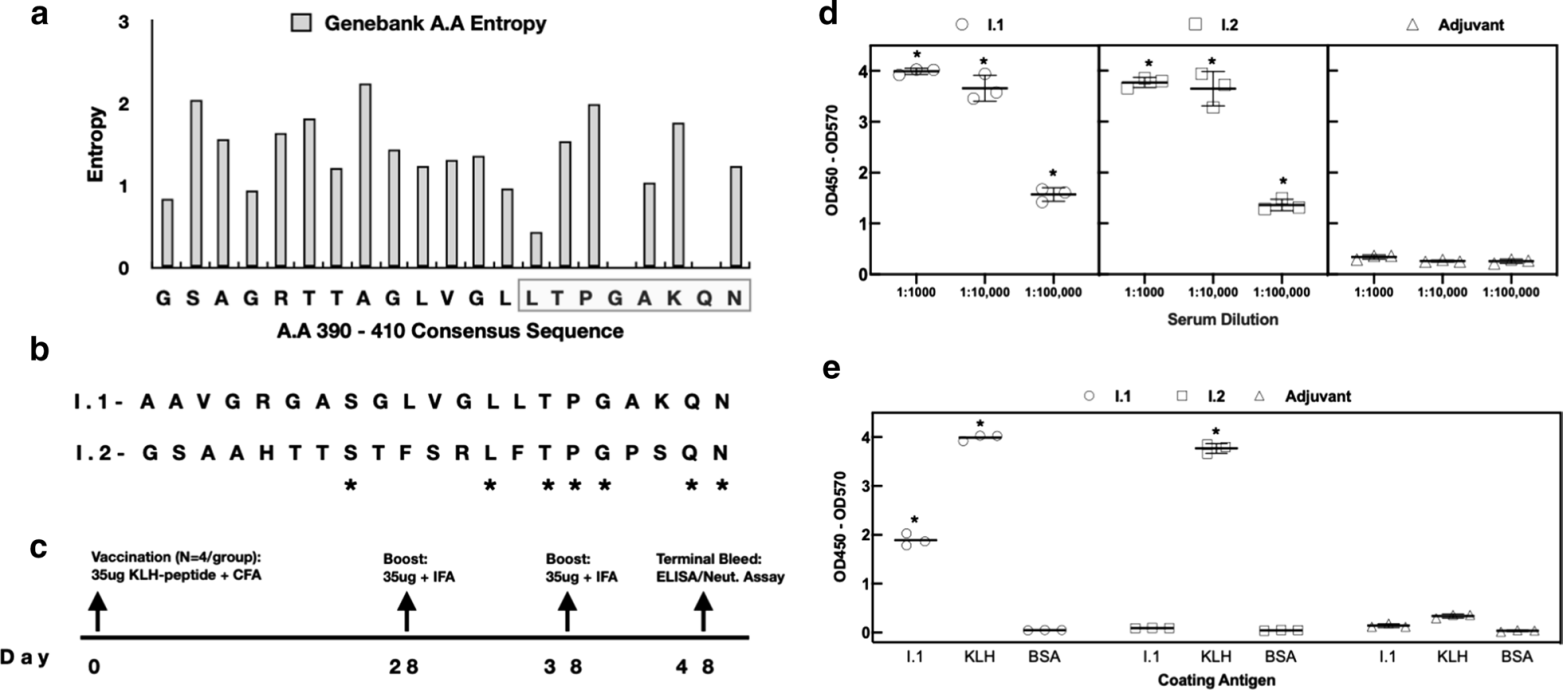
Monovalent vaccinations elicits high-titre, immunogen specific Ab. **a** HVR1 variability in a GenBank reference set was visualized by Shannon Entropy. Higher entropy corresponds to positions of greater variation. **b** Candidate immunogens (I.1, 1.2) differ at 14/21 amino acids. Conserved positions are indicated by asterisk. **c** Vaccination protocol for I.1 and 1.2 immunogens. Groups of 4, female Balb/C mice were vaccinated with either immunogen I.1, I.2, or adjuvant alone. Mice were bled on day 48, with group sera pooled for subsequent assays. **d** An ELISA plate was coated with immunogen I.1 (left panel), I.2 (middle panel), or both I.1/I.2 (right panel). Heat-inactivated mouse sera from each vaccine group was added at the indicated concentrations. **e** Separate rows of an ELISA plate were coated with either I.1-peptide (unconjugated), KLH (+), or BSA (−). Heat-inactivated mouse sera was added at 1:1000 dilution. For both ELISA, the binding of antibody was detected with anti-mouse secondary antibody. Averages of data from triplicates are shown. Statistical analysis was done by one-tailed, unpaired *t *test. *P < 0.001

HVR1 amino acid (AA) variability was visualized as Shannon Entropy using a GenBank reference set (Fig. 1a) [12]. Low entropy residues correspond to positions under purifying selection, and predominate in a putative C-terminal neutralizing epitope (Fig. 1a, blue shading) [6]. Using a patient derived clonal library encoding genotype 1a HVR1 sequences, high-Hamming distance (low pairwise sequence similarity) clones I.1 and I.2, differing at 14/21 AA, were selected for synthesis as 21-mer peptides (Fig. 1b). Peptides were then N-terminally conjugated to keyhole limpet hemocyanin (KLH) and adjuvanted with CFA for mouse immunizations (Fig. 1c). In both vaccine groups (I.1, I.2), we observed high-titre (1:100,000) immunogen specific Ab following vaccination (Fig. 1d). Sera from mock immunized mice (adjuvant only), were not reactive by ELISA at any dilution tested (Fig. 1d, right panel). Consistent with prior reports of the isolate-specificity of HVR1 targeting Ab, we did not observe cross-reactivity between the low sequence similarity I.1 and I.2 (Fig. 1e).

**Fig. 2:**
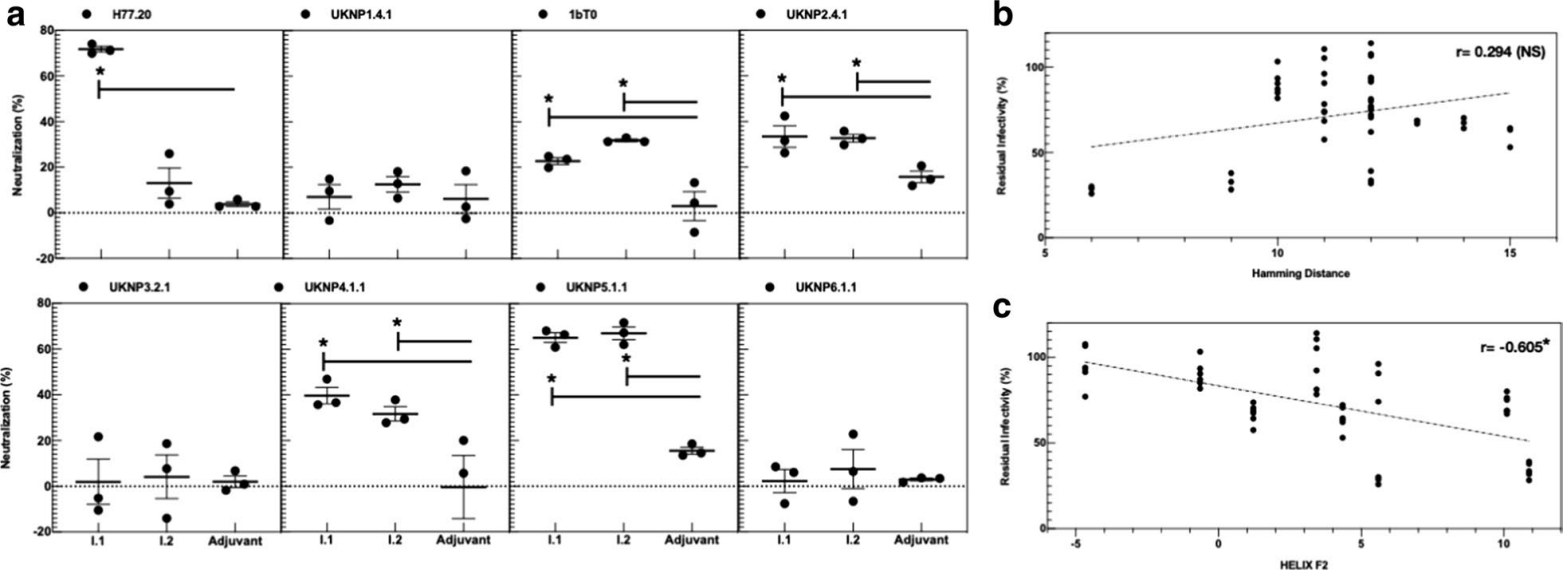
Monovalent vaccination elicits cross-nAb to low sequence similarity isolates. **a** Neutralization of HCVpp pseudotyped with H77.20 (1a), UKNP1.4.1 (1a), 1bTO (1b), UKNP2.4.1 (2a), UKNP3.2.1 (3a), UKNP4.1.1 (4a), UKNP5.1.1 (5a), and UKNP6.1.1 (6a) at 1:100 serum dilution. Neutralization was normalized to the infectivity of uninhibited virus. Statistical analysis was done by unpaired *t *test followed by FDR (Q = 0.05) adjustment for multiple comparison (*FDR-adjusted *P* < 0.05). Only significant differences were highlighted. **b** The residual infectivity of variants treated with vaccine sera was plotted against immunogen-virus Hamming distance. Hamming distance was calculated as the number of differing residues in the 21 AA immunogen alignment. **c** Residual infectivity was mapped against a secondary structure factor (HELIXF2), calculated for each aligned HVR1 sequence using the program CRASP. Statistical analysis was done using linear regression, *P < 0.05

We then examined if Ab elicited by immunization with either KLH-I.1 or KLH-I.2 were neutralizing using a multi-isolate panel of HCV-pseudotyped virus (HCVpp) representing genotypes 1–6 [17]. We observed cross-neutralization of HCVpp in both vaccine groups, with the infectivity of isolates from genotypes 1a, 1b, 2a, 4a, and 5a, significantly inhibited following incubation with antisera (Fig. 2a). Remarkably, despite low sequence similarity between the two immunizing peptides, similar patterns of neutralization were observed across isolates, implying an intrinsic HVR1 neutralization-sensitivity phenotype. This contrasts with prior reports that HVR1-based immune evasion is a property emergent from the host-specific Ab response [18]. Subsequent analysis confirmed that sequence similarity (Hamming distance) between vaccine immunogen and HCVpp isolate was not significantly associated with sensitivity to cross-neutralization (Fig. 2b). We then explored the relationship between sensitivity to neutralization and the integral physicochemical features of each isolates HVR1, such as aggregate propensity for α-helical configuration, which may directly influence Ab paratope:epitope affinity, using the secondary structure factor HELIXF2 [15]. A significant association was observed between secondary structure and neutralization sensitivity (Fig. 2c). That sensitivity to neutralization by vaccine elicited Ab was associated with intrinsic structural properties of the viral antigen, but not sequence similarity to the vaccine immunogen, further implies the existence of an intrinsic neutralization-sensitivity phenotype in HVR1. Intrinsic nAB sensitivity-phenotypes have been reported in conserved epitopes of HCV, and for HIV tier-2 variants, but to our knowledge this is the first report of neutralization sensitivity in a variable epitope reflecting integral physicochemical properties rather than similarity to vaccine immunogen [17]. Though tertiary structure, glycan shields, and E2-core domain epitopes may influence HVR1-mediated neutralization, and better predict neutralization sensitivity, the intrinsic disordering of HVR1 poses a challenge to structural models, which is reflected in our use of a multivariate analysis accounting only for features of secondary structure (HELIXF2) [10, 15].

## Conclusion

Our preliminary findings have critical implications for the development of a protective HCV vaccine. Given HVR1-mediated immune escape is operative in both natural infection and following vaccination, the possibility that certain HCV variants are resistant to neutralization by HVR1-targeting Ab, independent of immunogen or prior exposure, suggests that simply increasing the neutralization breadth of vaccine elicited Ab may be inadequate for protection from infection [18]. Considering HVR1 is the principal neutralizing-epitope, future studies investigating if particular immunogens, or immunogen combinations, can elicit HVR1-targeting Ab capable of neutralizing resistant variants is warranted. Our findings suggest that basing subsequent investigations on sequence similarity may be insufficient. We presented a crude alternative, using a measure of secondary structure, that appeared to better approximate sensitivity to neutralization. However, novel approaches more accurately accounting for the intrinsic structural features of distinct HVR1 variants are needed to resolve this long-standing challenge in HCV vaccine development.

## Data Availability

Sequences used to pseudotype HCVpp H77.20, 1.4.1, 2.4.1, 3.2.1, 4.1.1, 5.1.1, and are available in GenBank (Accession Nos. NC_038882, KU285161, KU285213, KU285218, KU285220, KU285225, KU285227). HVR1 sequences of 1bTO, I.1, I.2, are available upon request. Sequences used for NPJ constructing are available from (https://hcv.lanl.gov/content/sequence/NEWALIGN/align.html) with parameters “2008/E2/protein”, or from corresponding author upon request. Data and detailed protocol for ELISA and neutralization assays are available from author upon request.

## Abbreviations

HCV: Hepatitis C virus
HVR1: Hypervariable region 1
DAA: Direct acting antiviral
E2: Glycoprotein E2
nAb: Neutralizing antibody
AA: Amino acid
KLH: Keyhole limpet hemocyanin
CFA: Complete Freunds adjuvant
ELISA: Enzyme linked immunosorbent assay
HCVpp: HCV pseudovirus
